# The Neuroprotective Effects of Cyanidin Derivatives on AlCl_3_-Induced Zebrafish Model of Alzheimer’s Disease

**DOI:** 10.3390/molecules30183686

**Published:** 2025-09-10

**Authors:** Yun Wu, Yidan Gao, Fangfang Tie, Ruinan Wang, Na Hu, Qi Dong, Chunxiang Fu, Honglun Wang

**Affiliations:** 1 Qinghai Provincial Key Laboratory of Tibetan Medicine Research and CAS Key Laboratory of Tibetan Medicine Research, Northwest Institute of Plateau Biology, Xining 810008, China; wuyun@nwipb.cas.cn (Y.W.); gaoyidan@nwipb.cas.cn (Y.G.); fftie@nwipb.cas.cn (F.T.); wangruinan@nwipb.cas.cn (R.W.); huna@nwipb.cas.cn (N.H.); qdong@nwipb.cas.cn (Q.D.); fucx@qibebt.ac.cn (C.F.); 2 University of Chinese Academy of Sciences, Beijing 100049, China

**Keywords:** Alzheimer’s disease, acetylcholinesterase, aluminum, cyanidin, molecular docking, zebrafish

## Abstract

Alzheimer’s disease (AD) is characterized by cholinergic deficits and neuronal damage, making acetylcholinesterase (AChE) a crucial therapeutic target. Cyanidin derivatives, sourced from the diet as anthocyanins, exhibit neuroprotective properties, yet comparative investigations are scarce. This research explored the neuroprotective impacts of five cyanidin derivatives, namely cyanidin-3-*O*-(trans-p-coumaroyl)-diglycoside (C3GG), cyanidin-3-*O-*rutinoside (C3R), cyanidin-3-*O-*arabinoside (C3A), cyanidin-3-*O-*sophoroside (C3S), and cyanidin-3*-O-*xyloside (C3X), utilizing an aluminum-chloride (AlCl_3_)-induced zebrafish model of AD. The administration of these compounds ameliorated zebrafish locomotor impairments, suppressed AChE activity, decreased brain oxidative stress levels, upregulated AD-related gene expression, and mitigated brain tissue pathological changes. Molecular docking and dynamics simulations indicated that cyanidin derivatives exhibit robust binding affinity and stable binding to AChE. Particularly, C3R demonstrated the most potent multi-faceted neuroprotective effects among the tested derivatives, suggesting its potential as a promising lead compound for AD therapy.

## 1. Introduction

Alzheimer’s disease (AD) is a chronic and progressively debilitating neurodegenerative disorder characterized by memory loss, cognitive decline, behavioral disturbances, and impairment in activities of daily living [1]. The pathophysiology of AD is marked by the accumulation of β-amyloid (Aβ) plaques within neurons, which are closely associated with neuronal and synaptic deterioration, as well as dysfunction of the cholinergic neurotransmitter system [2]. The global prevalence of AD is steadily increasing. Projections indicate that by 2050, the number of individuals affected may exceed 153 million [3]. This trend poses substantial economic and healthcare challenges, particularly in aging populations [4]. While the exact pathogenesis of AD remains elusive, disruption of cholinergic neuronal activity has been identified as a potential causative factor [5]. In patients with AD, the elevated production of acetylcholinesterase (AChE) in the brain leads to reduced acetylcholine synthesis and impaired cortical cholinergic function [6]. Current AD treatments predominantly center on AChE inhibitors, which aim to enhance cholinergic transmission [7,8]. However, the therapeutic efficacy of these agents has been found to be limited. Consequently, developing drugs that can improve the function of the cholinergic system has become a critical direction in AD therapy [9,10].

Aluminum is recognized as one of the environmentally prevalent toxic elements and has been linked to cognitive dysfunction. It is considered a potential risk factor for AD [11]. In the presence of aluminum chloride (AlCl_3_) in the brain, this compound interacts with the cholinergic system, leading to disruptions in cholinergic neurotransmission and an elevation in AChE activity [12,13]. This interaction can result in increased degradation of acetylcholine [14], heightened amyloid production [15], impaired learning capabilities [16], memory deficits, and other hallmark symptoms associated with AD [17]. Zebrafish, serving as a valuable complement to rodent models, have increasingly been utilized in the study of neurological disorders and the development of neuropharmacological agents [18,19,20]. They play an indispensable role in establishing AD models [21,22,23]. Research has demonstrated that zebrafish larvae exposed to AlCl_3_ display behavioral and biochemical alterations comparable to those observed in patients with AD, such as impaired motor function, increased AChE activity, and heightened oxidative stress [24]. Accordingly, AlCl_3_ was administered in this study to develop an AD-like features in zebrafish [25].

Cyanidin, a prevalent anthocyanin, is a member of the flavonoid family, recognized for its bioactive properties. It primarily exists in nature as glycosylated or acylated derivatives, such as monoglycosides, diglycosides, and acylated or methylated forms. The catechol structure and multiple hydroxyl groups of cyanidin derivatives confer a wide range of pharmacological activities, including antioxidant [26], anti-inflammatory [27], neuroprotective [28], anti-aging [29], antidiabetic [30,31], and antitumor effects [32], highlighting their therapeutic potential. For instance, cyanidin-3-*O*-glucoside (C3G) has demonstrated the ability to protect neurons from cytotoxicity and functional impairments induced by various pathological factors [33,34]. Importantly, while the activity of common derivatives like C3G has been explored, a systematic comparative analysis of various glycosylated forms is lacking. Such a comparison is essential to establish a structure–activity relationship (SAR) and identify the most promising candidate for AD therapy.

Additionally, numerous studies have investigated the protective effects of anthocyanins and their derivatives, including cyanidins, in models of AD. For instance, a previous study reported the protective effects of anthocyanins against intracerebroventricular-streptozotocin (icv-STZ)-induced neurotoxicity, demonstrating that these compounds can alleviate cognitive impairment by modulating ion pump activity and cholinergic neurotransmission [35]. More recently, a systematic review further summarized the potential of anthocyanins in ameliorating AD-related pathologies and behavioral deficits, highlighting their multi-target mechanisms [36]. In addition, emerging evidence indicates that certain cyanidin derivatives can significantly inhibit AChE activity and reduce Aβ aggregation, further supporting their potential as therapeutic candidates for AD [37].

Nevertheless, the majority of current studies have primarily focused on a limited set of commonly studied derivatives, such as C3G, while systematic comparative analyses of other glycosylated forms—such as C3GG, C3R, C3A, and related compounds—remain limited. Therefore, this study was designed to directly compare the neuroprotective efficacy of five cyanidin derivatives with different sugar conjugates (C3GG, C3R, C3A, C3S, and C3X) using an AlCl_3_-induced zebrafish model of AD. A comprehensive methodological approach was employed, integrating in vivo behavioral, biochemical, transcriptional, and histological assessments, alongside in silico molecular docking and molecular dynamics simulations to investigate interactions with AChE. This integrative strategy not only identifies C3R as the most effective neuroprotective candidate among the tested derivatives but also offers key insights into its underlying mechanisms of action and potential limitations, thereby establishing a crucial basis for future optimization and development of cyanidin-based therapeutic agents for AD.

## 2. Results

### 2.1. The Effects of Cyanidin Derivatives on AlCl_3_-Induced Locomotor Deficits of Zebrafish Larvae

**Figure 1:**
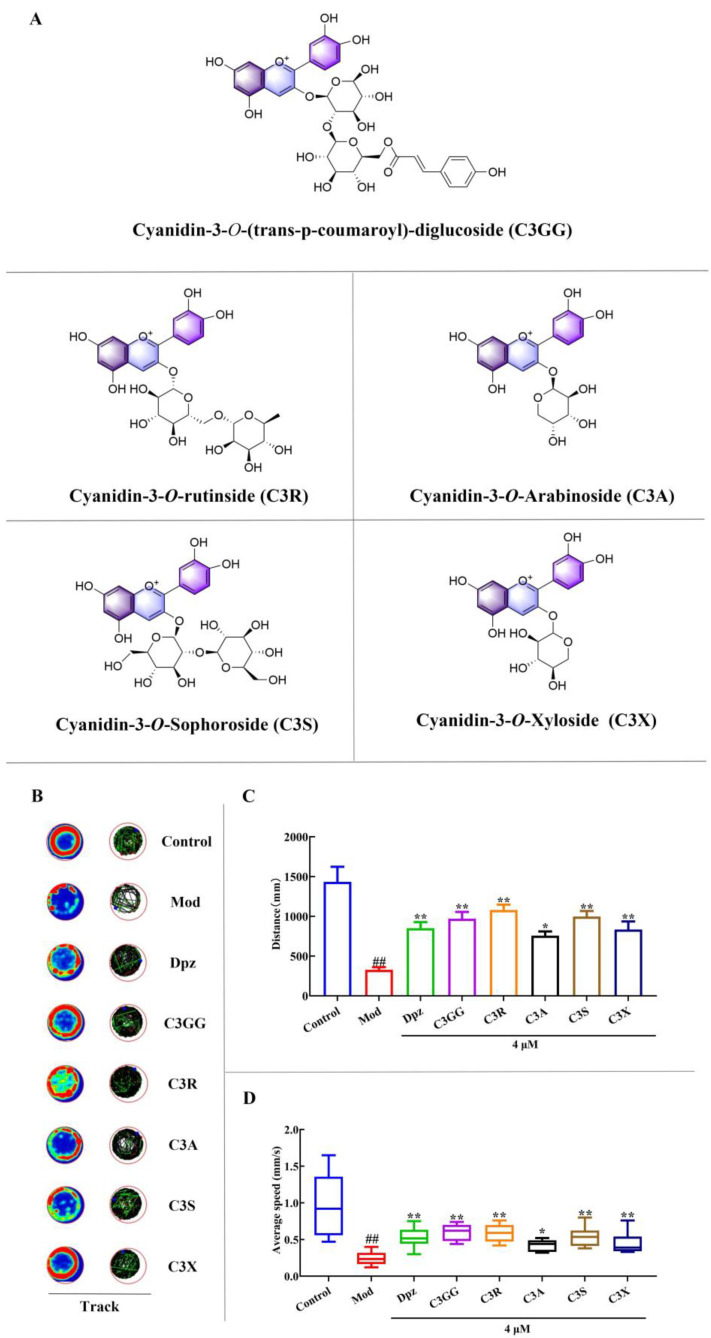
The effects of cyanidin derivatives on locomotor impairments of AD zebrafish. (**A**) The chemical structure and abbreviations of cyanidin derivatives. (**B**) A representative image of the larvae movement track from each group. In the heat map, the colors red, green, and blue are used to represent fast, medium-speed, and slow actions, respectively. (**C**) Distance travelled in mm. (**D**) Average speed in mm/s. ## *p* < 0.01 vs. Con group, * *p* < 0.05 and ** *p* < 0.01 vs. model group. Group notes: Control: control group, Mod: model group (AlCl_3_), Dpz: donepezil + AlCl_3_ group, C3GG: cyanidin-3-*O*-(trans-p-coumaroyl)-diglycoside + AlCl_3_ group, C3R: cyanidin-3-*O*-rutinoside + AlCl_3_ group, C3A: cyanidin-3-*O*-arabinoside + AlCl_3_ group, C3S: cyanidin-3-*O*-sophoroside, + AlCl_3_ group, C3X: cyanidin-3-*O*-xyloside+ AlCl_3_ group.

The locomotor behavior of zebrafish is closely associated with their neural development. To investigate the motor impairments induced by AlCl_3_ and the potential neuroprotective effects of cyanidin derivatives (Figure 1A), behavioral tests were performed on zebrafish larvae at 6 dpf. The representative swimming trajectories of the zebrafish are presented in Figure 1B. As shown in Figure 1, exposure to AlCl_3_ resulted in a significant decrease in both the total distance (Figure 1C) and average speed (Figure 1D) of free-swimming zebrafish compared to the control group (*p* < 0.01). The results illustrate that AlCl_3_ impairs the locomotor capacity of zebrafish. Consequently, the establishment of the zebrafish AD model was successful. Following treatment with 4 µM donepezil (Dpz) and cyanidin derivatives, there was an increase observed in both the distance traveled and the speed change of zebrafish compared to those in the AD group (*p* < 0.05, Figure 1C,D). The results suggest that Dpz improves dysfunction induced by AlCl_3_ in zebrafish, indicating its potential therapeutic effects. Interestingly, a similar trend of behavioral change was also observed in the cyanidin derivatives treatment groups. When zebrafish were co-treated with AlCl_3_ and different types of cyanidin derivatives, their dyskinesias were significantly reduced. These results demonstrate the effective alleviation of motor behavior impairment in zebrafish larvae caused by AlCl_3_ through the application of cyanidin derivatives, suggesting its potential as a protective agent against AD-like symptoms induced by AlCl_3_ in zebrafish.

### 2.2. The Effects of Cyanidin Derivatives on AlCl_3_-Induced the Dysfunction of Cholinergic System and Oxidative Stress

**Figure 2:**
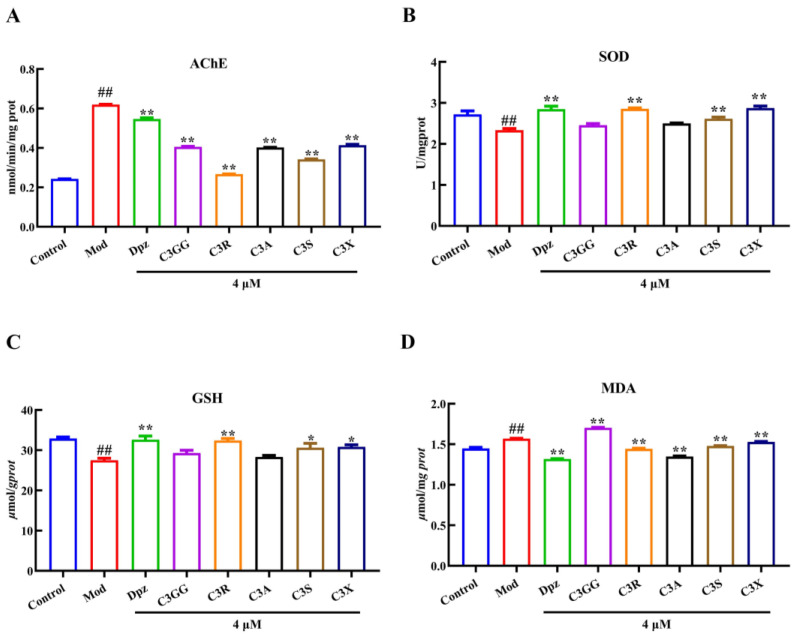
The effects of cyanidin derivatives on the dysfunction of cholinergic system and oxidative stress of AD zebrafish. (**A**) AChE activity. (**B**) SOD activity. (**C**) GSH level. (**D**) MDA level. ## *p* < 0.01 vs. Con group, * *p* < 0.05 and ** *p* < 0.01 vs. model group. Group notes: Control: control group, Mod: model group (AlCl_3_), Dpz: donepezil + AlCl_3_ group, C3GG: cyanidin-3-*O*-(trans-p-coumaroyl)-diglycoside + AlCl_3_ group, C3R: cyanidin-3-*O*-rutinoside + AlCl_3_ group, C3A: cyanidin-3-*O*-arabinoside + AlCl_3_ group, C3S: cyanidin-3-*O*-sophoroside, + AlCl_3_ group, C3X: cyanidin-3-*O*-xyloside+ AlCl_3_ group.

To investigate the efficacy of cyanidin derivatives treatment in alleviating choline dysfunction and oxidative stress in an AlCl_3_-induced AD model, we measured the activities of AChE, SOD, GSH, and MDA in zebrafish larvae. The activities of AChE in the groups are depicted in Figure 2A. AlCl_3_ significantly increased AChE activity compared to the control group. However, cyanidin derivatives and Dpz demonstrated partial restoration of these AlCl_3_-induced alterations in AChE activity (*p* < 0.01), aligning with the outcomes observed in behavioral assessments. The zebrafish exposed to AlCl_3_ demonstrated a significant increase in MDA levels and a reduction in GSH and SOD levels, as depicted in Figure 2B–D, when compared to the normal control group (*p* < 0.01). On the other hand, treatment with cyanidin derivatives and Dpz at a concentration of 4 µM significantly enhanced SOD enzymatic activity to a partial extent, elevated reduced GSH levels, and concurrently decreased MDA concentrations (Dpz, C3R, C3S and C3X; *p* < 0.05). The results demonstrated that cyanidin derivatives effectively mitigate AlCl_3_-induced cholinergic system dysfunction and oxidative stress in zebrafish by enhancing the activity of nerve conduction markers and antioxidant enzymes. In conclusion, cyanidin derivatives exhibit promising potential as anti-AD agents.

### 2.3. The Effects of Cyanidin Derivatives on the Expression of AD-Related Genes

**Figure 3:**
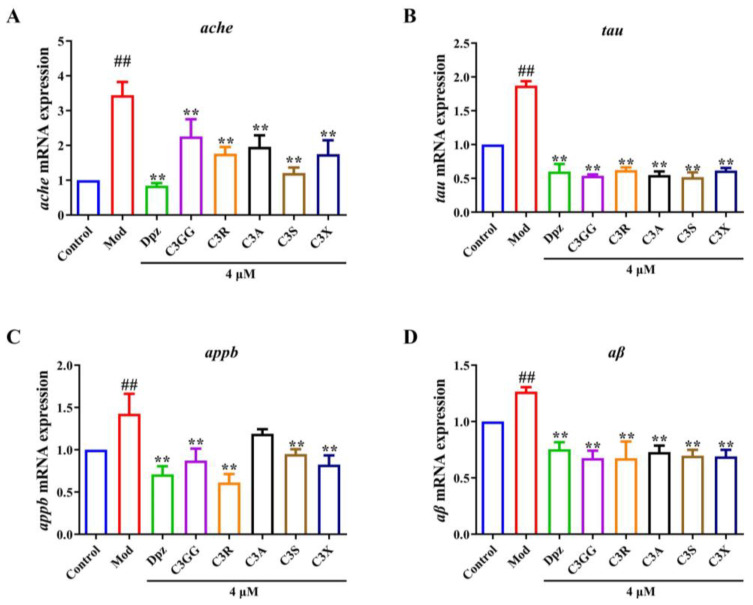
The effects of cyanidin derivatives on the transcriptional alterations of AD-related genes of AD zebrafish. (**A**) Relative mRNA expression of *ache*. (**B**) Relative mRNA expression of *tau*. (**C**) Relative mRNA expression of *appb*. (**D**) Relative mRNA expression of *aβ*. ## *p* < 0.01 vs. Con group, ** *p* < 0.01 vs. model group. Group notes: Control: control group, Mod: model group (AlCl_3_), Dpz: donepezil + AlCl_3_ group, C3GG: cyanidin-3-*O*-(trans-p-coumaroyl)-diglycoside + AlCl_3_ group, C3R: cyanidin-3-*O*-rutinoside + AlCl_3_ group, C3A: cyanidin-3-*O*-arabinoside + AlCl_3_ group, C3S: cyanidin-3-*O*-sophoroside, + AlCl_3_ group, C3X: cyanidin-3-*O*-xyloside+ AlCl_3_ group.

To evaluate the protective effects of cyanidin derivatives against AlCl_3_-induced symptoms resembling AD, we first investigated the expression of AD-related genes. The amyloid precursor protein (APP) acts as the precursor molecule for the Aβ peptide, a key pathological factor in AD [38]. AChE plays a crucial role in the detrimental feedback loop involving Aβ and phosphorylated tau proteins, with diverse isoforms of these proteins identified in cholinergic neurons located in the basal forebrain system [39]. Elevated AChE activity intensifies cholinergic deficits and promotes the formation of Aβ fibrils, thereby playing a significant role in the development of AD [40]. Changes in the expression or activity of these genes have the potential to trigger the degeneration of neurons within the basal forebrain cholinergic system. The expression levels of *ache*, *tau*, *appb*, and *aβ* were significantly upregulated (*p* < 0.01) in zebrafish larvae exposed to AlCl_3_ (Figure 3A–D). However, treatment with cyanidin derivatives reversed these increases (*p* < 0.01, excepted C3A). These findings suggest that cyanidin derivatives have the potential to alleviate AD-like symptoms in zebrafish. The qRT-PCR results further suggest that AlCl_3_ seriously caused cholinergic damage in zebrafish, while cyanidin derivatives treatment significantly alleviated the adverse effects.

### 2.4. The Protect Effects of Cyanidin Derivatives on AlCl_3_-Induced Histopathological Lesions in the Larval Brain

**Figure 4:**
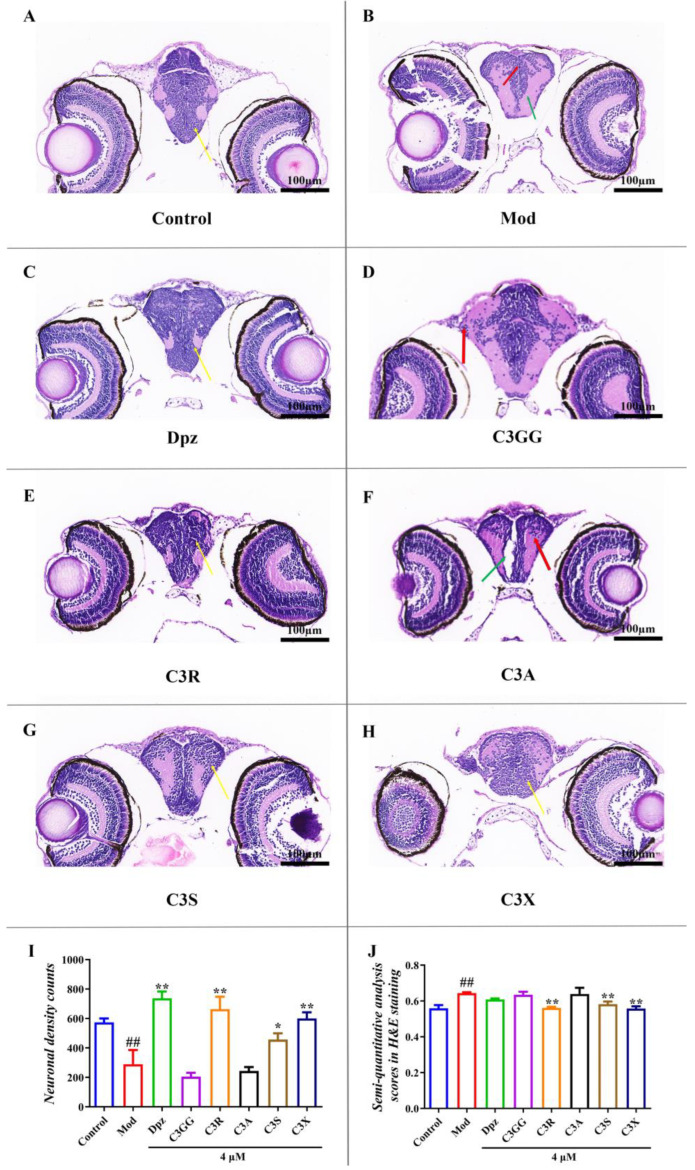
Comparison of histopathological alterations in brain tissues among various treatment groups. (**A**) Normal histological section of larval brain (40×). (**B**) Larvae brain exposed to 80 μM AlCl_3_ (40×). (**C**) Larvae brain exposed to AlCl_3_ + 4μM Dpz (40×). (**D**) Larvae brain exposed to AlCl_3_ + 4μM C3GG (40×). (**E**) Larvae brain exposed to AlCl_3_ + 4μM C3R (40×). (**F**) Larvae brain exposed to AlCl_3_ + 4μM C3A (40×). (**G**) Larvae brain exposed to AlCl_3_ + 4μM C3S (40×). (**H**) Larvae brain exposed to AlCl_3_ + 4μM C3X (40×). (**I**) Neuronal density in zebrafish brain. (**J**) semi-quantitative analysis scores in H&E staining. ## *p* < 0.01 vs. Con group, * *p* < 0.05 and ** *p* < 0.01 vs. model group. (The neurons are highlighted by yellow arrows. Red arrows indicate a small population of neurons exhibiting pyknosis and deeply stained necrosis, whereas green arrows point to areas of midbrain dilation accompanied by balloon-like degeneration).

The H&E staining results demonstrated that in the normal group, the brain tissue exhibited a normal and intact overall structure (Figure 4A). Moreover, the midbrain displayed an abundance of neatly arranged neurons, characterized by robust morphology with spherical or slightly oval nuclei. Conversely, zebrafish brain sections treated with AlCl_3_ revealed severe ballooning degeneration and pyknotic neurons exhibiting dark staining without a visible nucleus or nucleolus (Figure 4B). Additionally, there was defective organization observed in the mesencephalon region, along with a few shrunken and sickle-shaped cells. When treated with cyanidin derivatives and Dpz, these compounds demonstrate the ability to significantly reduce the number of abnormal neurons in the zebrafish brain, enhance the proportion of healthy neurons, and effectively alleviate cellular damage, neuronal edema, and necrosis (Figure 4C–H). It is noteworthy that C3R, C3S, and C3X effectively reversed the pathological alterations caused by AlCl_3_ exposure. No morphological abnormalities were detected in the brains of zebrafish, and the quantity, morphology, and structural integrity of neuronal cells were markedly restored. These findings indicate that the three cyanidin derivatives exhibit substantial therapeutic potential against aluminum-chloride-induced neuronal degeneration (Figure 4I,J).

**Figure 5:**
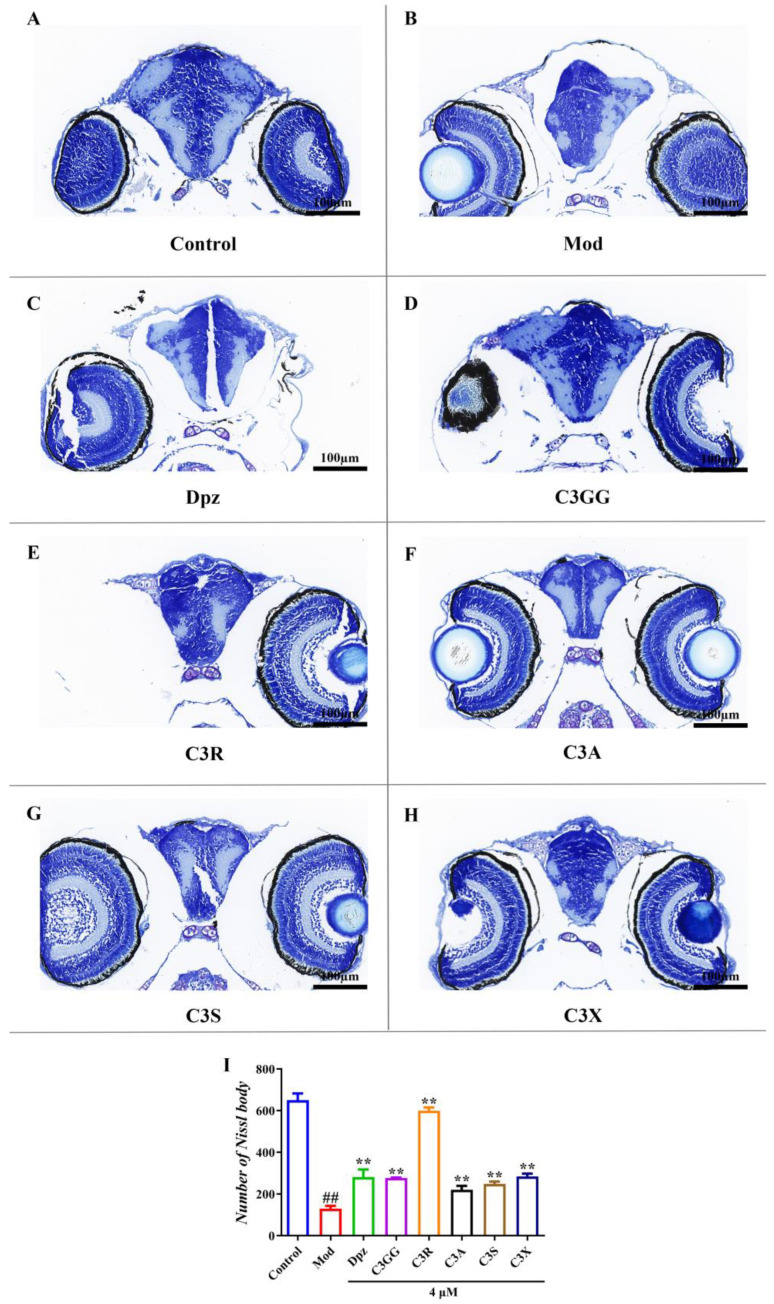
Comparison of Nissl staining of neural cells in brain tissues among various treatment groups. Group notes: Control: control group, Mod: model group (AlCl_3_), Dpz: donepezil + AlCl_3_ group, C3GG: cyanidin-3-*O*-(trans-p-coumaroyl)-diglycoside + AlCl_3_ group, C3R: cyanidin-3-*O*-rutinoside + AlCl_3_ group, C3A: cyanidin-3-*O*-arabinoside + AlCl_3_ group, C3S: cyanidin-3-*O*-sophoroside, + AlCl_3_ group, C3X: cyanidin-3-*O*-xyloside+ AlCl_3_ group. (**A**) Nissl staining diagram of the normal group (40×). (**B**) Nissl staining diagram of the model group (40×). (**C**) Nissl staining diagram of the Dpz group (40×). (**D**) Nissl staining diagram of the C3GG group (40×). (**E**) Nissl staining diagram of the C3R group (40×). (**F**) Nissl staining diagram of the C3A group (40×). (**G**) Nissl staining diagram of the C3S group (40×). (**H**) Nissl staining diagram of the C3X group (40×). (**I**) The number of Nissl bodies in the zebrafish brain. ## *p* < 0.01 vs. Con group, ** *p* < 0.01 vs. model group.

Furthermore, Nissl staining was employed to evaluate neuronal loss across all experimental groups. A consistent deep-blue staining intensity was used as the standardized criterion for assessing all positive images, which were then quantitatively analyzed. In the control group, neurons appeared morphologically intact and well-structured, with a high density of Nissl bodies (34.36%). In contrast, the AlCl_3_-treated group exhibited a marked reduction in Nissl bodies (13.28%), along with cellular damage, disorganization, reduced cell size, and cytoplasmic condensation or absence of staining. Notably, treatment with cyanidin derivatives and Dpz significantly attenuated neuronal damage (Figure 5C–H), resulting in a considerable increase in the number of Nissl bodies and an improved Nissl body ratio. The Nissl body ratios were as follows, Dpz (30.91%), C3GG (9.72%), C3R (36.94%), C3A (14.22%), C3S (17.97%), and C3X (20.46%). Following treatment with the five cyanidin derivatives or Dpz, a significant increase in the Nissl body count was observed in the brains of AlCl_3_-exposed AD zebrafish (# *p* < 0.01, Figure 5I), accompanied by notable improvements in neuronal morphology and cellular architecture. These findings on neuroprotective effects align with the results of behavioral and biochemical assays and are further supported by histopathological observations from H&E staining. Collectively, these results provide strong evidence that cyanidin derivatives can significantly ameliorate AlCl_3_-induced neuronal damage in zebrafish, with C3R demonstrating the most robust neuroprotective effect (Figure 5I).

### 2.5. Molecular Docking Results

**Figure 6:**
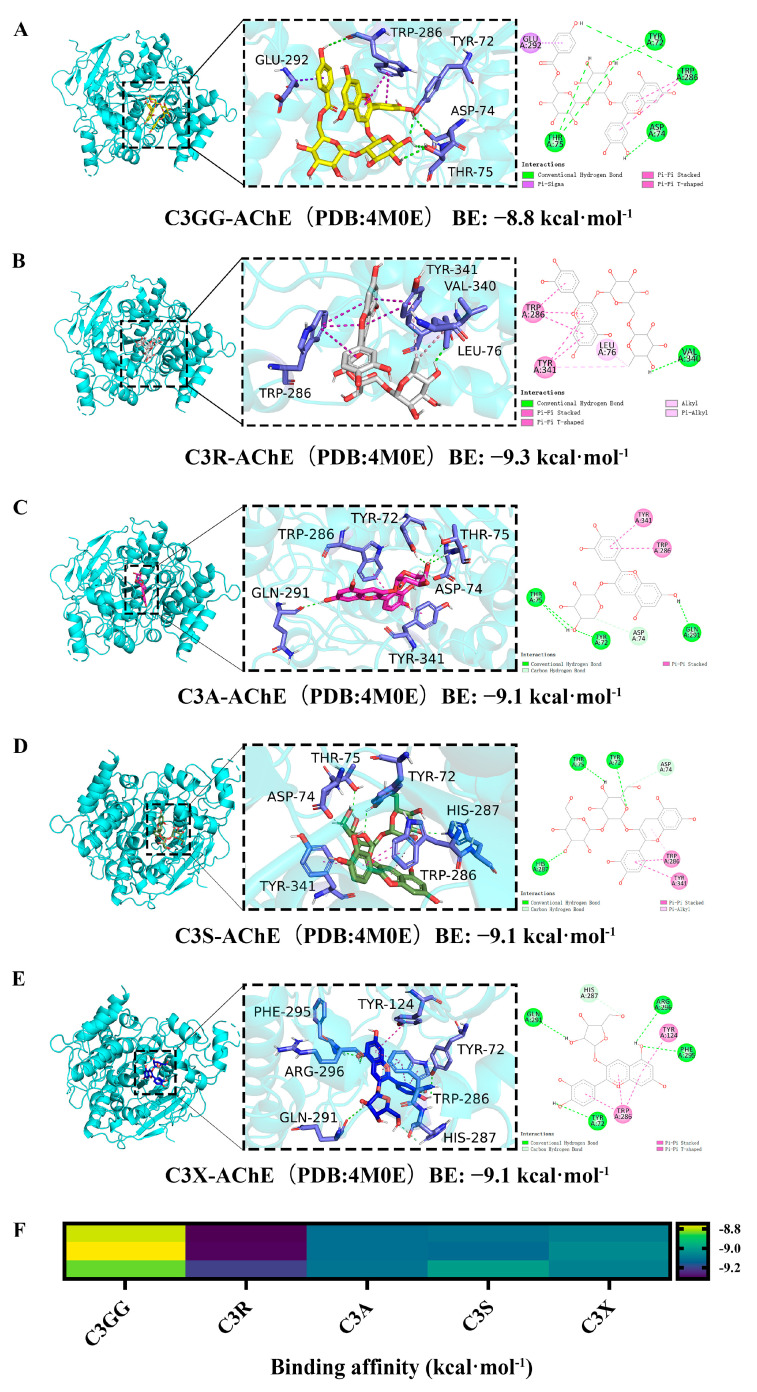
Molecular docking results of AChE and cyanidin derivatives. (**A**) C3GG. (**B**) C3R. (**C**) C3A. (**D**) C3S. (**E**) C3X. (**F**) Binding affinity between AChE and cyanidin derivatives.

The previous studies have indicated that AChE inhibitors effectively treat AD by interacting with AChE [41]. The binding model and affinity between cyanidin derivatives and the crystallographic structures of AChE protein are depicted in Figure 6. The interaction forces between the cyanidin derivatives and potential targets of AChE primarily consist of polar interactions (H-Bonds) and non-polar interactions (Figure 6A–E). Molecular docking analysis demonstrated that all cyanidin derivatives exhibit potential binding to the active site of AChE with high affinity, as evidenced by docking scores ranging from −8.8 to −9.3 kcal/mol. These scores are comparable to that of the positive control, Dpz, which had a docking score of −9.2 kcal/mol (Supplementary Figure S1). Although the docking scores of C3R, C3A, C3S, and C3X were closely similar, the binding affinity of C3R was slightly higher, at −9.3 kcal/mol (Figure 6B). However, considering the limitations of docking scores in distinguishing highly similar ligands, more accurate binding free energy calculations were conducted using MD simulations combined with MM/GBSA analysis.

### 2.6. MD Simulation Results

**Figure 7:**
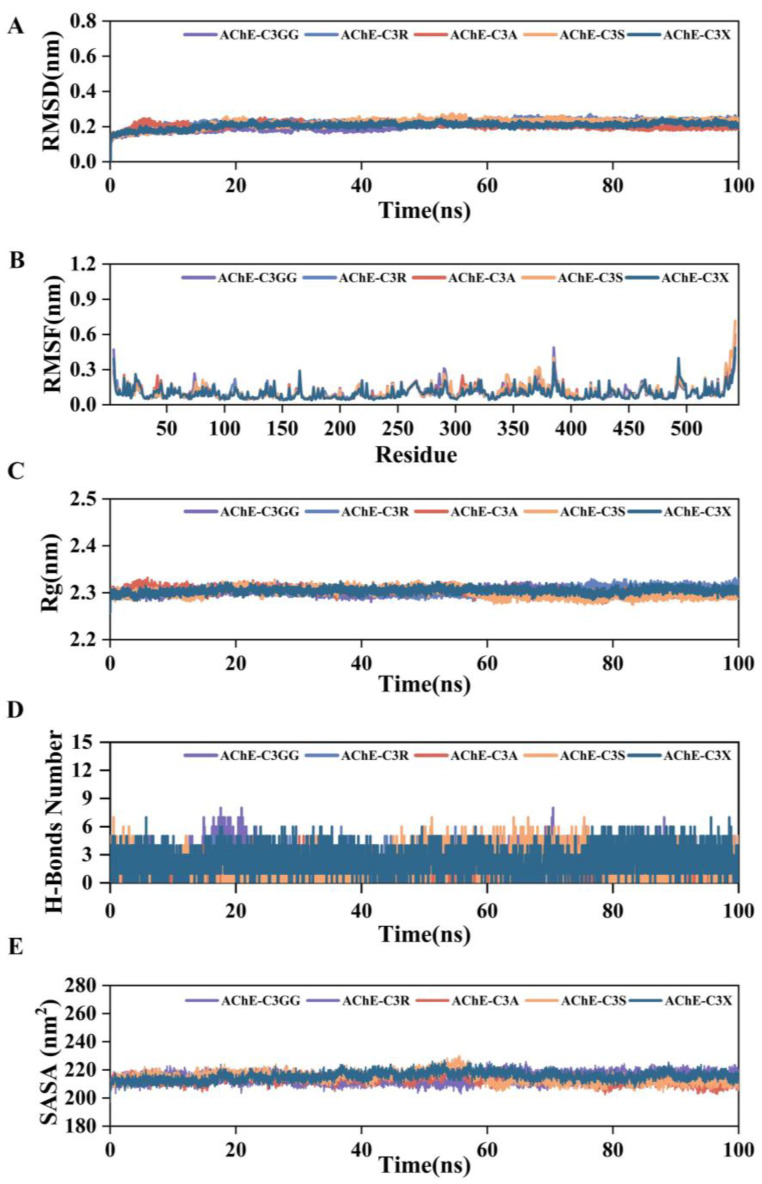
Visualization of results from molecular dynamics simulations. (**A**) RMSD. (**B**) RMSF. (**C**) Rg. (**D**) H-Bonds Number. (**E**) SASA.

**Figure 8:**
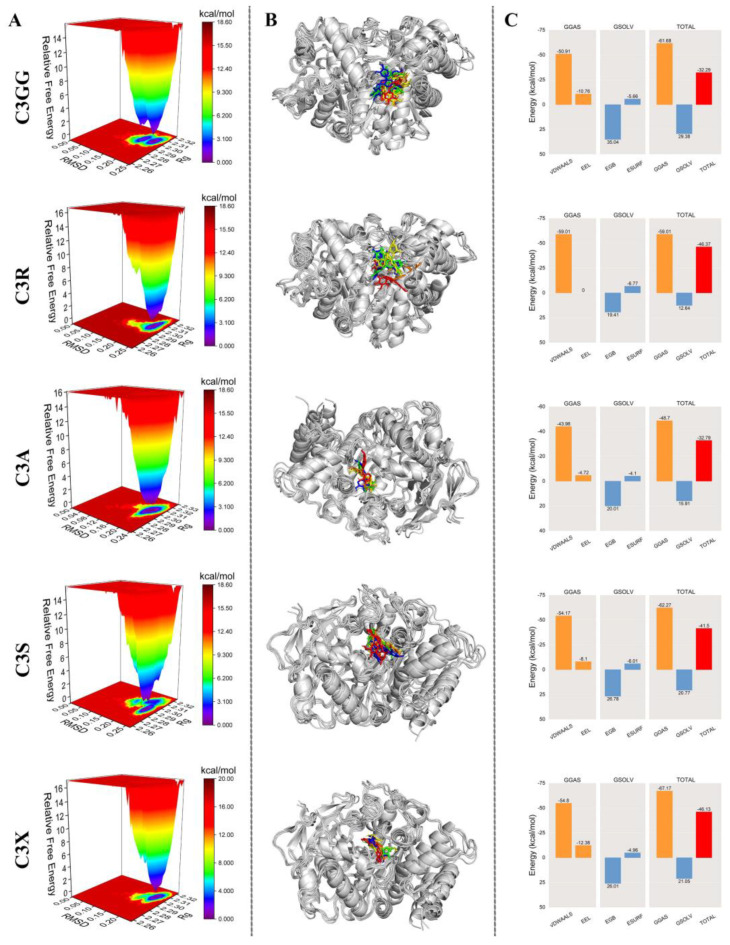
Molecular dynamics simulation results. (**A**) The free energy landscape map of cyanidin derivatives. (**B**) Structural changes of the AChE-cyanidin derivatives complex at different time points. The colors red, green, blue, yellow, and orange, respectively, represent the structural comparisons of molecular dynamics simulations at 0, 25, 50, 75, and 100 ns. (**C**) Free energy of cyanidin derivatives binding to AChE calculated by MM/GBSA method. VDWAALS, EEL, EGB, ESURF, GGAS, GSOLV, and TOTAL represent van der Waals forces, electrostatic potential energy, polar solvation energy, nonpolar solvation energy, molecular mechanics term, solvation energy term, and binding free energy, respectively.

To validate the docking results, we analyzed the MD simulation trajectories of AChE protein complexed with cyanidin derivatives. We examined the RMSD, RMSF, Rg, and the number of HBonds, SASA, relative free energy distribution, and structural comparisons of the complexes at 0, 25, 50, 75, and 100 ns. In addition, we used the MM/GBSA method to calculate the average binding free energy between the proteins and ligands. As shown in Figure 7A–C, the fluctuation ranges of RMSD, RMSF, and Rg values of the complexes of AChE and cyanidin derivatives were small, and they remained stable throughout the simulation process, indicating that they were formed of the complexes was well stabilized. The number of H-Bonds formed between the AChE protein and cyanidin derivatives remained consistently stable, indicating effective stabilization of the complexes (Figure 7D). Additionally, the SASA curves of the AChE-cyanidin derivatives complexes exhibited stable fluctuations throughout the simulation, suggesting that cyanidin derivatives exerted minimal influence on the overall structure of the protein (Figure 7E). We further analyzed the relative free energy distribution and comparison of the complex structures as shown in Figure 8A; the complexes formed between AChE protein and cyanidin derivatives were well stabilized in the free energy distribution. As illustrated in Figure 8B, the results indicate that cyanidin derivatives form more stable complexes with the AChE protein, whereas C3GG and C3S form fewer stable complexes with the AChE protein. To quantitatively compare the binding affinities, we calculated the binding free energy using the MM/GBSA method. The average binding free energies were calculated to be −32.29 (C3GG), −46.37 (C3R), −32.79 (C3A), −41.50 (C3S), and −46.13 (C3X) kcal/mol (Figure 8C). This analysis revealed that C3R and C3X formed the most stable complexes with AChE, exhibiting significantly more favorable binding free energies than C3GG and C3A. C3S displayed an intermediate binding affinity. These results provide a more robust computational basis for the observed in vivo efficacy ranking.

### 2.7. Prediction of ADMET and Drug-Likeness Properties

The ADMET and drug-likeness properties of the cyanidin derivatives were predicted using ADMETlab 2.0, with the results provided in Supplementary Table S2. The analysis revealed a combination of advantageous and challenging characteristics. On the one hand, all compounds exhibited favorable MDCK permeability, blood–brain barrier penetration, and a low predicted risk of inhibiting key cytochrome P450 enzymes (CYP2C19, CYP2C9, CYP2D6, and CYP3A4), suggesting a reduced potential for drug–drug interactions. Moreover, most compounds showed a low probability of hERG inhibition (indicating a low cardiotoxicity risk) and human hepatotoxicity (H-HT). Furthermore, these compounds satisfied one or more drug-likeness criteria such as MCE-18 [42] and the Pfizer rule [43], indicating certain advantageous pharmacological properties. On the other hand, the predictions also highlighted several limitations of the cyanidin derivatives. These include a predicted risk of drug-induced liver injury (DILI) and a high probability of skin sensitization. Additionally, their drug-likeness may be suboptimal, as only the smaller molecular weight derivatives C3A and C3X complied with Lipinski’s rule, while most compounds failed the GSK rule. These predictions suggest that, although the cyanidin derivatives possess encouraging biological activities and meet certain criteria, they may face limitations in oral bioavailability and carry toxicity risks that warrant further investigation.

## 3. Discussion

The diminished functionality of cholinergic neurons represents a hallmark characteristic of AD [5], as evidenced by elevated AChE activity. Increased AChE activation exacerbates oxidative stress [44]. One therapeutic approach to enhance cholinergic neurotransmission entails augmenting acetylcholine levels via the inhibition of AChE [45]. To investigate the neuroprotective effects of cyanidin derivatives, we examined changes in AChE activity in zebrafish larvae. Our results demonstrated that AlCl_3_ treatment increased AChE activity, which subsequently reduced ACh levels and impaired neurotransmission, leading to cognitive decline. This finding is consistent with previous studies by Sun et al. [46]. Cyanidin derivatives was found to reduce AChE activity, thereby enhancing learning and memory functions. Previous reports have indicated that anthocyanins and flavonoids can act as AChE inhibitors [47,48,49], and our study confirms that cyanidin derivatives possesses similar inhibitory properties. Consistent with our findings, Mohamed et al. [50] demonstrated that raspberry ketone inhibits the brain’s AChE and consequently increases acetylcholine levels.

*AChE* is the gene encoding AChE, which inactivates the neurotransmitter ACh by catalyzing its hydrolysis into choline and acetate. In this study, we observed that AlCl_3_ treatment significantly increased AChE mRNA expression, while cyanidin derivatives mitigated this increase. Furthermore, cyanidin derivatives also obviously reduced the mRNA expression of *tau*, *appb*, and *aβ*, findings consistent with those reported by Suresh et al. [51]. The U.S. FDA has approved ChEIs, such as Dpz, galantamine, and rivastigmine, for the treatment of AD [52]. We utilized Dpz as a positive control to evaluate the efficacy of cyanidin derivatives on an AlCl_3_-induced zebrafish model of AD. The results demonstrated that cyanidin derivatives, when tested at 4 µM, exhibited comparable or even superior efficacy relative to Dpz. Notably, the five cyanidin derivatives demonstrated significant therapeutic effects in the model, suggesting their potential as valuable lead compounds for the development of anti-AD drugs. It should be noted that the findings of this study are primarily based on transcriptional evidence. The lack of protein-level data (e.g., Western blot) for key targets like AChE, due to the current unavailability of specific antibodies for zebrafish, remains a limitation that will be addressed in future research.

To further investigate the binding affinity of cyanidin derivatives to AChE, molecular docking simulations were conducted to evaluate the interaction between cyanidin derivatives and AChE. The simulations revealed strong hydrogen bonding and π–π interactions between cyanidin derivatives and the active site of AChE, suggesting a rapid onset of action and supporting its potential to alleviate AD symptoms through AChE inhibition. Additionally, molecular mechanics simulations were performed to assess the degree and stability of binding between various cyanidin derivatives and AChE. The results indicated that five cyanidin derivatives exhibited excellent binding stability with regard to the AChE protein, with C3R demonstrating the strongest binding affinity. A recent study identified malvidin-3-O-galactoside (M3G) as a potent AChE binder, with a binding free energy of −71.546 kJ/mol (−17.1 kcal/mol) involving key residues GLU-118 and TYR-408 [53]. Our research on C3R revealed a binding free energy of −46.37 kcal/mol using MM/GBSA. The disparity in binding energies likely arises from differences in computational methods (MMPBSA vs. MM/GBSA), ligand structure, and binding mode. Unlike M3G, C3R interacts with AChE via multiple forces, including a hydrogen bond with VAL-340, alkyl/π–alkyl interactions with LEU-76, and π–π stacking with TRP-286 and TYR-341. The disaccharide rutinoside in C3R, compared to the monosaccharide galactoside in M3G, may also affect solvation and conformational entropy. These results highlight how structural nuances in anthocyanins, such as aglycone type and glycosylation pattern, significantly influence their interaction with AChE. Our study further explores cyanidin derivatives and provides in vivo validation of C3R’s efficacy, reinforcing its potential as a lead compound for AD therapy.

Our ADMET predictions provide a nuanced perspective on the therapeutic potential of cyanidin derivatives. While certain favorable properties were observed—including BBB permeability, which is critical for effective AD treatment [54], and a low cytochrome P450 inhibition profile—several limitations are also evident. These include a high predicted risk of drug-induced liver injury and skin sensitization. Moreover, the failure to comply with key drug-likeness rules such as Lipinski’s and GSK’s suggests possible challenges in oral absorption and overall pharmacokinetics. It should be noted, however, that ADMET properties are derived from in silico predictions and are not fully conclusive. Therefore, further pharmacological and toxicological studies remain essential to fully evaluate the promising potential of these cyanidin derivatives in the treatment of AD.

The neuroprotective efficacy of C3R likely stems from its distinctive disaccharide structure. Rutinoside, a disaccharide, is composed of glucose and rhamnose linked by an α-1,6 bond [55]. Rutinoside (glucose–rhamnose), in comparison to sophoroside (glucose–glucose) in C3S or monosaccharides present in other derivatives, may offer advantages in terms of enhanced water solubility. This characteristic renders it more soluble in physiological environments than monosaccharide glycosides such as C3G, thereby promoting better absorption. Additionally, its balanced polarity and lipophilic properties facilitate its ability to cross the BBB. Additionally, the rhamnose terminal of rutinoside may establish additional hydrogen bonds with the active site of AChE, while the flexible glycosidic linkage of the disaccharide allows for favorable adaptation to the allosteric pocket of AChE. In contrast, the bulkier acylated diglycoside (C3GG) may exhibit reduced brain uptake, whereas the simpler monoglycosides (C3A) may lack the necessary binding stability or favorable pharmacokinetic profile. The central finding of this study arises from the direct comparison of five cyanidin derivatives, which revealed significant differences in their neuroprotective efficacy. Specifically, C3R and C3X demonstrated substantial therapeutic potential, providing compelling evidence to support their further development as valuable lead compounds for the treatment of AD. These results facilitate the formulation of a preliminary SAR for this class of compounds.

Excessive exposure to aluminum triggers an inflammatory response in the central nervous system, leading to increased AChE activity and oxidative stress in the brain [56]. Previous studies have demonstrated that aluminum exposure during the larval stage of zebrafish induces oxidative stress in the brain and results in behavioral abnormalities [25,57]. These findings were corroborated in the present study, where cyanidin derivatives significantly mitigated AlCl_3_-induced oxidative stress by enhancing GSH levels, increasing SOD enzymatic activity, and reducing MDA content. This indicates that cyanidin derivatives exerts a protective effect against AlCl_3_-induced oxidative stress through the enhancement of the antioxidant defense system. Therefore, enhancing the activity of the antioxidant defense system within the body is one of the key mechanisms by which anthocyanin derivatives exert their neuroprotective effects.

Dyskinesia serves as a critical parameter for assessing the severity of AD [58]. Motor function is fundamental to the survival of zebrafish, and diminished swimming speed in these organisms is a key indicator of cognitive decline and aging [59]. In this study, upon stimulation, zebrafish larvae in the AlCl_3_ treatment group exhibited markedly reduced swimming speeds, reflecting cognitive impairment. However, following cyanidin derivatives treatment, the motor deficits observed in the AD zebrafish model were significantly ameliorated. These findings indicate that cyanidin derivatives may possess potential therapeutic benefits for AD.

In addition to inducing behavioral alterations, exposure to AlCl_3_ results in significant pathological changes in brain tissue [60]. AlCl_3_ leads to severe histopathological abnormalities and markedly disrupts cellular morphology, including extensive cell death and neuronal damage [61]. However, treatment with cyanidin derivatives and Dpz has effectively mitigated these histopathological changes. Specifically, AlCl_3_-induced vacuolization and neuronal cell loss observed in Nissl-stained sections were attenuated in the group treated with cyanidin derivatives and Dpz, leading to an increase in the number of Nissl bodies. Overall, cyanidin derivatives exhibit the capability to mitigate nerve damage, enhance neuronal proliferation, and preserve neuronal integrity at both behavioral and tissue levels. It is important to note that the AlCl_3_-induced model primarily mimics the cholinergic deficit and oxidative stress components of AD, rather than the full amyloid and tau pathology. While it serves as a valuable tool for preliminary screening, further validation in more complex models—such as adult zebrafish or transgenic zebrafish expressing human APP or tau—is necessary to confirm the therapeutic potential of the most effective candidates (C3R, C3S, and C3X). In conclusion, through the application of a comparative approach, this study provides novel insights into the SAR of cyanidin derivatives in the context of AD treatment.

Collectively, this study indicates that cyanidin derivatives act as potent effective neuroprotective compounds, highlighting their potential therapeutic application in the intervention of AD. Further research is warranted to fully elucidate the molecular mechanisms underlying their neuroprotective effects.

## 4. Materials and Methods

### 4.1. Chemicals and Reagents

AlCl_3_·6H_2_O was purchased from Sigma-Aldrich (St. Louis, MO, USA). Dpz and Eugenol was purchased from Shanghai McLean Biochemical Technology Co., Ltd. (Shanghai, China). C3GG is the main compound in *Nitraria tangutorum* Bobr, which was isolated according to the former method in our laboratory [62]. C3R, C3A, C3S, and C3X were procured from Shanghai Yuanye Biotechnology Co., Ltd. (Shanghai, China). Phosphate buffered saline (PBS) was purchased from Wuhan Pnousai Life Science Co., Ltd. (Wuhan, China). The Zebrafish AChE kit was provided by Shanghai Enzyme linked Biotechnology Co., Ltd. (Shanghai, China). Superoxide dismutase (SOD) and glutathione (GSH) assay kits were acquired from the Nanjing Jiancheng Bioengineering Institute (Nanjing, China). The bicinchoninic acid (BCA) and malondialdehyde (MDA) assay kits were purchased from Beyotime Biotechnology Co., Ltd. (Shanghai, China). The trizol and reverse transcription kit was obtained from TaKaRa Biotechnology Co., Ltd. (Dalian, China). ChamQ Universal SYBR qPCR Master Mix was purchased from Vazyme Biotech Co., Ltd. (Nanjing, China). Primers were synthesized by Shanghai Bioengineering Co., Ltd. (Shanghai, China).

### 4.2. Animals

The AB line zebrafish (*Danio rerio*) were acquired from Shanghai FishBio Co., Ltd., (Shanghai, China), and maintained in a zebrafish facility (Beijing Aisheng Technology Development Co., Ltd., Beijing, China) at 26 ± 2 °C, pH 6.0–8.0, and conductivity 500–700 μS/cm, with a 14 h light/10 h dark cycle photoperiod. The larvae were obtained through natural mating. This study utilized zebrafish larvae at 3 days post-fertilization (dpf), and they were cultured in E3 medium (Hubei Chuangxin Biotechnology Co., Ltd., Wuhan, China) at a constant temperature of 28.5 °C. All zebrafish experiments were conducted in compliance with the Chinese Academy of Sciences, the Northwest Institute of Plateau Biology of Animal Ethics Committee. The approval number for animal ethics is 2024-07.

### 4.3. Establishment of Zebrafish AD Model and Grouping

The larvae AD model was established by treating 3 dpf larvae zebrafish to 6 dpf with 80 µM AlCl_3_ [46]. The zebrafish larvae experiment was conducted as follows: healthy and normally developing zebrafish larvae at 3 dpf were meticulously selected and placed into six-well plates, with each well containing a group of 40 larvae, and this setup was replicated in three additional wells. The larvae were subsequently divided into several experimental groups: the control group (exposed to standard system water), the model group (treated with 80 μM AlCl_3_), the model + Dpz group (treated with 80 μM AlCl_3_ + 4 μM Dpz), and the model + cyanidin derivatives groups (treated with 80 μM AlCl_3_ + 4 μM cyanidin derivatives). The concentration (4 µM) of all compounds was determined based on a preliminary dose-finding experiment conducted with Dpz, which demonstrated that 4 µM was the lowest effective concentration capable of producing significant therapeutic effects in both behavioral and biochemical assays (Supplementary Figure S2).

### 4.4. Behavioral Analysis

Following a 3-day period of drug administration, twelve larvae were randomly selected from each group and rinsed with the E3 embryo medium. The zebrafish larvae cultured until 6 dpf were individually placed in 96-well culture plates containing 300 μL of E3 medium per well. The culture temperature was maintained at 28.5 °C. The zebrafish larvae in the 96-well plate were subjected to a 10 min acclimatization period in the instrument. All experiments were conducted within a 30 min timeframe, consisting of three cycles of alternating light and dark phases (each lasting for 5 min). VisuTrack AI 3.0 software (Shanghai Xinruan Information Technology Co., Ltd., Shanghai, China) was used to record locomotor activity for each larva and analyze their movement distance and average speed.

### 4.5. Determination of Biochemical Indicator

After conducting behavioral tests, the zebrafish larvae were euthanized using Eugenol and subsequently rinsed three times with 1 × PBS. All excess water was removed, and the specimens were immediately stored in liquid nitrogen. Next, physiological saline (1:9 Mass/volume) was added to the samples, which were then mechanically homogenized under ice-cooling conditions. The resulting mixture was centrifuged at 12,000 rpm for 15 min at 4 °C. Following centrifugation, the supernatants were collected for further analysis. The protein concentration of each group was determined using the BCA method. Subsequently, we conducted the remaining steps of the assay following the instructions provided in the kit to determine AChE, SOD, GSH, and MDA activity in zebrafish specimens as indicators of the effectiveness of anti-AD compounds.

### 4.6. Detection of Gene Expression

Real-time fluorescence quantitative PCR (qRT-PCR) was employed to evaluate the expression levels of four genes associated with AD, namely *ache*, *tau*, *appb*, and *aβ* in zebrafish larvae. Following the completion of behavioral tests, the zebrafish larvae were humanely euthanized using a Eugenol solution and subsequently rinsed three times with 1 × PBS to remove any residual water. Each set of 20 larvae was then stored in individual 2 mL EP tubes for future experimentation. Total RNA extraction from each sample was performed using Trizol, ensuring that the OD_260/280_ ratios exceeded 1.8. The extracted RNA samples were reverse transcribed into cDNA following the manufacturer’s instructions provided with the cDNA synthesis kit. Quantitative PCR was conducted using the SYBR Green I method, with reactions performed in triplicate. The mean value of the triplicates was used for analysis. To normalize the results, β-actin expression served as an internal reference gene, and quantification was carried out using the comparative (2^−ΔΔCt^) method. A list of primers utilized in qRT-PCR is provided in Supplementary Table S1.

### 4.7. Hematoxylin-Eosin Staining (H&E) Staining and Nissl Staining

Histopathological studies were conducted using brain tissue of larvae zebrafish. Zebrafish larvae at 6 dpf were collected and fixed in 4% (*w*/*v*) paraformaldehyde overnight following the exposure experiment. Subsequently, the tissues underwent a series of ethanol dehydration steps, paraffin embedding, and slicing into longitudinal sections with a thickness of 5 μm. The tissue sections were then subjected to hematoxylin and eosin as well as Nissl staining. Following this, deparaffinization in xylene, alcohol dehydration, and coverage with neutral gum were performed. The telencephalic neuronal alterations were observed under a magnification of 40×. The number of neurons and Nissl bodies in the stained sections was quantitatively analyzed using Image-Pro Plus 6.0 software (software Media Cybernetics, Inc., Rockville, MD, USA).

### 4.8. Molecular Docking

The 3D structure of the receptor protein AChE (PDB ID: 4m0e) was obtained from the RCSB protein data bank (PDB) (https://www1.rcsb.org/ (accessed on 3 March 2025)). The 3D structure file of the small molecule compound cyanidin derivatives was downloaded from the PubChem database (https://pubchem.ncbi.nlm.nih.gov/ (accessed on 3 March 2025)), and Openbabel 3.1.1 was employed for molecular force field optimization to obtain the optimal molecular structure. The proteins were subjected to hydrogenation using AutoDock Tools 1.5.6. The Grid plate parameters were configured to define the docking range, while the semi-flexible docking mode was employed. Molecular docking was performed using AutoDock Vina 1.2.0 software with Lamarckian genetic algorithm as the selected method, enabling the acquisition of both binding free energy and docking result files. The docking results were visualized using PyMOL 2.3.0 software in conjunction with Discovery Studio 2019 (version 19.1, Accelrys Inc., San Diego, CA, USA, 2019). The PDB files of the complexes of proteins with small molecule compounds are provided in the Supplementary Materials.

### 4.9. Molecular Dynamics Simulation

The protein–ligand complexes obtained by molecular docking were subjected to MD simulations using Gromacs 2022.4 software. Amber14sb force was chosen as the protein force field, Gaff2 was applied for ligand parameterization, and the TIP4P water model was chosen to add solvents to the protein–ligand system. Prior to formal dynamics simulations, the complexes were subjected to 50,000 steps of energy minimization using a conjugate gradient algorithm, followed by further equilibration of the system at 100 ps using an isothermal (310 K) system (NVT) and an isobaric (1 standard atmospheric pressure) system (NPT) with a time step of 2 fs. Finally, unconstrained MD simulations were conducted for 100 ns at ambient temperature and pressure. The root means square deviation (RMSD), root mean square fluctuation (RMSF), radius of gyration (Rg), and number of hydrogen bonds (H-Bonds) between proteins and ligands were analyzed. The MM/GBSA method was utilized to calculate the average binding free energy between proteins and ligands.

### 4.10. In Silico Prediction of ADMET and Drug-Likeness Properties of Cyanidin Derivatives

Computerized ADMET evaluation models have been recognized as essential tools in the drug development process, significantly enhancing drug design and the optimization of lead compounds. In this study, we utilized ADMET lab 2.0 (https://admetmesh.scbdd.com/pub/ (accessed on 4 March 2025)) to comprehensively assess the pharmacokinetic profile and toxicity of cyanidin derivatives. This online platform offers robust predictions of a compound’s ADMET properties (Absorption, Distribution, Metabolism, Elimination, and Toxicity) and its drug-likeness characteristics [63].

### 4.11. Statistical Analysis

The data were presented as the mean ± standard error of mean (SEM) obtained from a minimum of three independent experiments. Statistical analysis was conducted using one-way analysis of variance (ANOVA), followed by Tukey’s honestly significant difference (HSD) post hoc test for multiple comparisons. All statistical analyses were performed using GraphPad Prism 9.4 software (GraphPad Software Inc., La Jolla, CA, USA). A *p*-value < 0.05 was considered statistically significant.

## 5. Conclusions

The findings of this study demonstrate that cyanidin derivatives exhibit potent neuroprotective effects in zebrafish models of AD. Cyanidin derivatives ameliorated abnormal behaviors, reduced oxidative stress levels, inhibited AChE activity, and downregulated the expression of AD-related genes. Molecular docking and dynamics simulations revealed that the cyanidin derivatives have a strong binding affinity for AChE, forming stable complexes. These neuroprotective effects are attributed to the inhibition of AChE activity and the antioxidant properties of the cyanidin derivatives, with the effects of C3R being particularly pronounced.

## Data Availability

Data are contained within the article and Supplementary Materials.

## Supplementary Materials

The following supporting information can be downloaded at: https://www.mdpi.com/article/10.3390/molecules30183686/s1, Figure S1: Molecular docking results of AChE and Dpz; Figure S2: Determination of the optimal concentration for donepezil (Dpz) in the AlCl_3_-induced zebrafish AD model; Table S1: Primer sequences used in present study; Table S2: ADMET and drug-likeness properties of five cyanidin compounds through online prediction tool of ADMET lab 2.0. File S1: Protein-ligand structures in PDB data.

## Abbreviations

The following abbreviations are used in this manuscript:

AChE	Acetylcholinesterase
AD	Alzheimer’s disease
ADMET	Absorption, Distribution, Metabolism, Elimination, Toxicity
AlCl_3_	Aluminum chloride
APP	amyloid precursor protein
BBB	Blood-brain barrier
BCA	Bicinchoninic acid
C3A	Cyanidin-3-*O*-arabinoside
C3GG	Cyanidin-3-*O*-(trans-p-coumaroyl)-diglucoside
C3R	Cyanidin-3*O*-rutinoside
C3S	Cyanidin-3-*O*-sophoroside
C3X	Cyanidin-3-*O*-xyloside
DILI	Drug-Induced Liver Injury
dpf	Days post-fertilization
Dpz	donepezi
GSH	Glutathione
H-Bonds	hydrogen bonds
H-HT	Human hepatotoxicity
MDA	Malondialdehyde
MDCK	Madin-Darby Canine Kidney cell
PBS	Phosphate buffered saline
PDB	Protein data bank
qRT-PCR	Real-time fluorescence quantitative PCR
RMSD	Root mean square deviation
RMSF	Root mean square fluctuation
Rg	Radius of gyration
SASA	solvent accessible surface area
SAR	structure-activity relationship
SOD	Superoxide dismutase
