# Supplementary material for: The Neuroprotective Effects of Cyanidin Derivatives on AlCl3-Induced Zebrafish Model of Alzheimer’s Disease
Source: Molecules. 2025 Sep 10;30(18):3686. doi: 10.3390/molecules30183686 (PMC12472917; doi:10.3390/molecules30183686)
Supplement: Supplementary file 1 [file molecules-30-03686-s001.zip › Supplementary materials.pdf]

Supplementary materials

The Neuroprotective Effects of Cyanidin Derivatives on $AlCl_3$-Induced Zebrafish Model of Alzheimer's Disease

Yun Wu [1,2], Yidan Gao [1,2], Fangfang Tie [1], Ruinan Wang [1,2], Na Hu [1], Qi Dong [1], Chunxiang Fu [1] and Honglun Wang [1,2,*]

1 Qinghai Provincial Key Laboratory of Tibetan Medicine Research and CAS Key Laboratory of Tibetan Medicine Research, Northwest Institute of Plateau Biology, Xining 810008, China; wuyun@nwipb.cas.cn (Y.W.); gaoyidan@nwipb.cas.cn (Y.G.); fftie@nwipb.cas.cn (F.T.); wangruinan@nwipb.cas.cn (R.W.); huna@nwipb.cas.cn (N.H.); qdong@nwipb.cas.cn (Q.D.); fucx@qibebt.ac.cn (C.F.)

2 University of Chinese Academy of Sciences, Beijing 100049, China

* Correspondence: hlwang@nwipb.cas.cn; Tel.: +86-13997384106

Table S1. Primer sequences used in present study

Gene	Sequence of the primer (5′- 3′)
β-actin	Forward: AGGTCATCACCATTGGCAAT
	Reverse: GATGTCCACGTCGCACTTCAT
ache	Forward: GGGCACCAGGTTTCAGTAAA
	Reverse: CTTTTGGCCGTTGTTCTCAT
tau	Forward: ACTGAGTTCTGGGAGCGAAA
	Reverse: CAAATGGGAATGTTGCTGTG
appb	Forward: GGTGACGGTGAAGATGATG
	Reverse: TCGCACAACCTCCTCTATG
aβ	Forward: AGCAGCTGGTGGAGACTCAT
	Reverse: TAGTGGCGGATAGTGTGCTG

Table S2 ADMET and drug-likeness properties of five cyanidin compounds through online prediction tool of ADMET lab 2.0.

	C3GG		C3R		C3A		C3S		C3X	
**Property**	**Value**	**Decision**	**Value**	**Decision**	**Value**	**Decision**	**Value**	**Decision**	**Value**	**Decision**
**Absorption**										
Caco-2 Permeability (log cm/s)	-6.669	🔴	-6.533	🔴	-6.313	🔴	-6.628	🔴	-6.237	🔴
MDCK Permeability (cm/s)	8e-06	🟢	3.3e-05	🟢	6e-06	🟢	4.2e-05	🟢	6.1e-05	🟢
Pgp-inhibitor	0.016	🟢	0.007	🟢	0.005	🟢	0.005	🟢	0.002	🟢
Pgp-substrate	0.994	🔴	0.999	🔴	0.997	🔴	0.996	🔴	0.997	🔴
HIA	0.859	🔴	0.953	🔴	0.063	🟢	0.923	🔴	0.968	🔴
$F_{20\%}$	0.105	🟢	0.594	🟡	0.887	🔴	0.033	🟢	1.0	🔴
$F_{30\%}$	1.0	🔴	0.999	🔴	0.972	🔴	1.0	🔴	0.998	🔴
**Distribution**										
PPB	94.51%	🔴	87.01%	🟢	90.28%	🔴	87.42%	🟢	92.80%	🔴
VD (L/kg)	0.54	🟢	0.384	🟢	0.564	🟢	0.353	🟢	0.712	🟢
BBB penetration	0.048	🟢	0.064	🟢	0.04	🟢	0.111	🟢	0.085	🟢
Fu	10.58%	🟢	12.39%	🟢	6.807%	🟢	15.63%	🟢	6.775%	🟢
**Metabolism**										
CYP1A2–inhibitor	0.208		0.118		0.152		0.021		0.43	
CYP1A2–substrate	0.011		0.024		0.041		0.025		0.026	
CYP2C19–inhibitor	0.05		0.033		0.027		0.012		0.122	
CYP2C19–substrate	0.041		0.052		0.049		0.055		0.055	
CYP2C9–inhibitor	0.033		0.018		0.136		0.002		0.432	
CYP2C9–substrate	0.215		0.077		0.168		0.24		0.159	
CYP2D6–inhibitor	0.22		0.038		0.117		0.005		0.429	
CYP2D6–substrate	0.149		0.124		0.169		0.124		0.108	
CYP3A4–inhibitor	0.052		0.029		0.078		0.043		0.179	
CYP3A4–substrate	0.003		0.013		0.068		0.013		0.061	

	C3GG		C3R		C3A		C3S		C3X	
**Property**	**Value**	**Decision**	**Value**	**Decision**	**Value**	**Decision**	**Value**	**Decision**	**Value**	**Decision**
**Excretion**										
CL (mL/min/kg)	4.661	🔴	1.899	🔴	9.245	🟢	0.959	🔴	1.425	🔴
$T_{1/2}$	0.784	-	0.799	-	0.881	-	0.824	-	0.802	-
**Toxicity**										
hERG blockers	0.059	🟢	0.028	🟢	0.019	🟢	0.056	🟢	0.003	🟢
H-HT	0.106	🟢	0.126	🟢	0.139	🟢	0.248	🟢	0.978	🔴
DILI	0.977	🔴	0.694	🟡	0.528	🟡	0.941	🔴	0.769	🔴
Ames toxicity	0.63	🟡	0.522	🟡	0.634	🟡	0.667	🟡	0.644	🟡
Rat oral acute toxicity	0.028	🟢	0.171	🟢	0.202	🟢	0.431	🟡	0.978	🔴
FDAMDD	0.006	🟢	0.009	🟢	0.038	🟢	0.052	🟢	0.98	🔴
Skin sensitization	0.92	🔴	0.953	🔴	0.945	🔴	0.952	🔴	0.94	🔴
Carcinogencity	0.158	🟢	0.048	🟢	0.046	🟢	0.225	🟢	0.14	🟢
Eye corrosion	0.003	🟢	0.003	🟢	0.003	🟢	0.003	🟢	0.003	🟢
Eye Irritation	0.63	🟡	0.191	🟢	0.607	🟡	0.269	🟢	0.052	🟢
Respiratory Toxicity	0.009	🟢	0.033	🟢	0.039	🟢	0.042	🟢	0.151	🟢
**Drug-likeness**										
MCE-18	138.182	🟢	120.7	🟢	86.538	🟢	120.7	🟢	84.615	🟢
Lipinski rule	rejected	🔴	rejected	🔴	accepted	🟢	rejected	🔴	accepted	🟢
Pfizer rule	accepted	🟢	accepted	🟢	accepted	🟢	accepted	🟢	accepted	🟢
GSK rule	rejected	🔴	rejected	🔴	rejected	🔴	rejected	🔴	rejected	🔴
Golden triangle	rejected	🔴	rejected	🔴	accepted	🟢	rejected	🔴	accepted	🟢

PS: 🟢 Excellent, 🟡 medium, 🔴 bad

Figure S1. Molecular docking results of AChE and Dpz

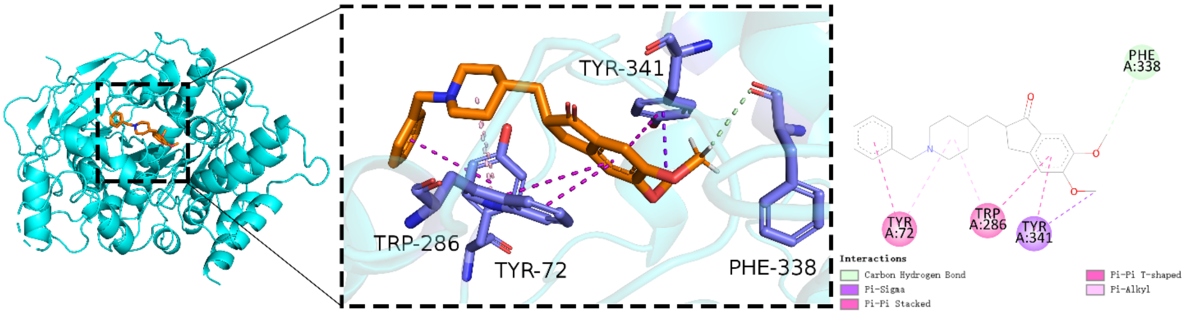

Donepezil -AChE（PDB:4M0E）BE:-9.2 (kcal·mol^{-1})

Figure S2. Determination of the optimal concentration for donepezil (Dpz) in the $AlCl_3$-induced zebrafish AD model

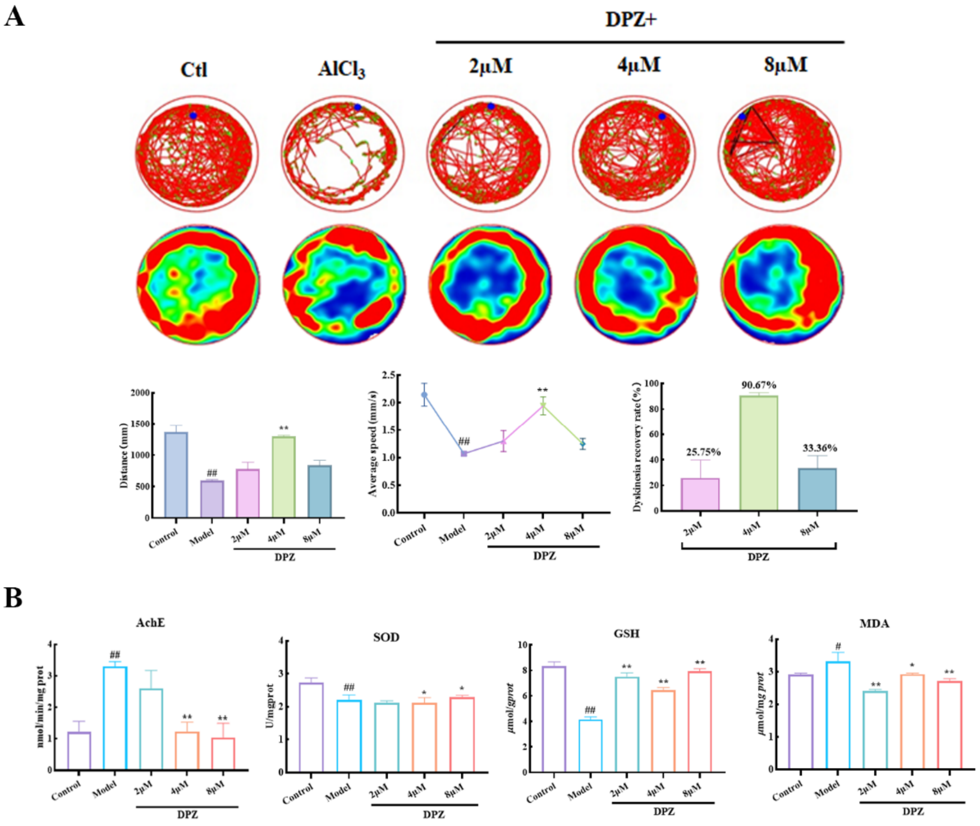

A The effects of Dpz on locomotor impairments of AD zebrafish. B. The effects of Dpz on the dysfunction of cholinergic system and oxidative stress of AD zebrafish. $^{\#}P < 0.05$ and $^{\#\#}P < 0.01$ vs Con group, $^{*}P < 0.05$ and $^{**}P < 0.01$ vs model group.
